# Focusing Resource Allocation-Wellbeing as a Tool for Prioritizing Interventions for Communities at Risk

**DOI:** 10.3390/ijerph10083435

**Published:** 2013-08-06

**Authors:** Anthony Hogan, Robert Tanton, Stewart Lockie, Sarah May

**Affiliations:** 1 The Australian and New Zealand School of Government’s Institute for Governance, University of Canberra, University Drive South, Canberra ACT 2601, Australia; 2 School of Sociology, The Australian National University, Canberra 0200, Australia; E-Mails: Stewart.Lockie@anu.edu.au (S.L.); Sarah.May@anu.edu.au (S.M.); 3 The National Centre for Social and Economic Modelling, University of Canberra, University Drive South, Canberra ACT 2601, Australia; E-Mail: Robert.Tanton@natsem.canberra.edu.au

**Keywords:** vulnerability, climate variability and change, resilience, adaptive capacity, social capital, thresholds in wellbeing

## Abstract

*Objective*: This study examined whether a wellbeing approach to resilience and adaptation would provide practical insights for prioritizing support to communities experiencing environmental and socio-economic stressors. *Methods*: A cross-sectional survey, based on a purposive sample of 2,196 stakeholders (landholders, hobby farmers, town resident and change agents) from three irrigation-dependent communities in Australia’s Murray-Darling Basin. Respondents’ adaptive capacity and wellbeing (individual and collective adaptive capacity, subjective wellbeing, social support, community connectivity, community leadership, in the context of known life stressors) were examined using chi-square, comparison of mean scores, hierarchical regression and factor-cluster analysis. *Results*: Statistically significant correlations (*p* < 0.05) were observed between individual (0.331) and collective (0.318) adaptive capacity and wellbeing. Taking into account respondents’ self-assessed health and socio-economic circumstances, perceptions of individual (15%) and collective adaptive capacity (10%) as well as community connectivity (13%) were associated with wellbeing (R^2^ = 0.36; F (9, 2099) = 132.9; *p* < 0.001). Cluster analysis found that 11% of respondents were particularly vulnerable, reporting below average scores on all indicators, with 56% of these reporting below threshold scores on subjective wellbeing. *Conclusions*: Addressing the capacity of individuals to work with others and to adapt to change, serve as important strategies in maintaining wellbeing in communities under stress. The human impacts of exogenous stressors appear to manifest themselves in poorer health outcomes; addressing primary stressors may in turn aid wellbeing. Longitudinal studies are indicated to verify these findings. Wellbeing may serve as a useful and parsimonious proxy measure for resilience and adaptive capacity.

## 1. Introduction

As humans are increasingly exposed to new and sustained stressors from the physical and socio-economic environments, policy-makers and community leaders are concerned as to the extent to which humans are susceptible to some form of collapse in the face of such stressors. Building on the work of ecologists, resilience in turn has come to be the new buzzword in policy and research as rural communities work towards developing a sustainable future [1,2,3,4,5,6]. Without doubt, these communities are having to deal with substantive stressors on a frequent basis. Indeed, the one constant facing agri-dependent communities in Australia’s Murray-Darling Basin (locally referred to as Australia’s *food bowl*) is the ongoing impact of change. Change has resulted from a variety of factors including the longer term impacts of market liberalization [7], disruption to the structure of rural life (either as a result of rural decline, or social displacement as a result of resource booms) [8,9], climate change and variability including drought, periodic fire storms and flooding [10,11,12,13], degradation of the river systems, changing rights to irrigated water [14], solostalgia [15], loss of biodiversity [14] and localised policy developments [16,17]. Extensive work has in turn been undertaken on the resilience of people in the face of external stressors, focusing on their capacity to adapt to change. Indeed adaptation is seen as being central to managing and minimizing any impacts of more intensified climate variability and longer term climate change [18]. Key insights from the literature note the importance of adaptive capacity with regard to peoples’ resilience [19,20] and capacity for transformative action [21,22,23]. Following from this research is the increasing desire to identify potential tipping points in human wellbeing and to identify strategies which communities can use to buffer themselves and be resilient against the impacts of such shocks [24].

The potential for a relationship to exist between exogenous stressors (environmental; socio-economic) and human wellbeing is implicit in various studies on adaptive capacity [25,26]. However, the nature and extent of this relationship has not been well developed in the literature, particularly within rural settings [27,28]. The literature links wellbeing (as an outcome where life needs were satisfied) with adaptive capacity, but notably at a community or systems level [29]. Cork *et al.* [29] challenged this notion though, by theorizing that wellbeing and adaptive capacity may not necessarily be the specific attributes of communities as a whole, but that such attributes may be distributed within such communities. Cork *et al.* [29] undertook an extensive review of the literature on quality of life and wellbeing, with a view to identifying the most appropriate method for measuring wellbeing, and particularly with a view to identifying a method that could help identify potential tipping points in human wellbeing. They identified that within the Australian context, Cummins’ [30] index of subjective wellbeing (SWB) was the most useful tool presently available which could be used to examine the sustainability of human wellbeing in the face of known stressors. The index offers a method for assessing “an individual’s global evaluation of his or her life across a variety of different aspects of life” [30]. Cummins’ [31] work on subjective wellbeing centers on the idea of individuals being satisfied with key domains of their everyday life (e.g., work, housing, relationships, future security), taking into account the inter-relationship between social capital, susceptibility and psycho-social wellbeing [30]. Central to Cummins’ [30] model is the insight that satisfaction with life reflects the capacity of the individual not just to cope with but to have positively adapted to their life circumstances and challenges. In the face of shorter term and significant life stressors, he argued that over time people can bounce back or be resilient. Cummins has proposed that wellbeing in humans is homeostatic in nature such that individuals can sustain their sense of subjective wellbeing over time in the face of specific stressors, up to a given point. However, he and his colleagues also argued that a threshold point exists with regard to the intensity, and potentially, duration of persistent stressors, and that when this point is passed, individual wellbeing breaks down. 

Importantly, the homeostatic nature of wellbeing means that, over time, even after a significant stressor, most people will return to their normal level of subjective wellbeing. Without doubt, there are some conceptual challenges associated with Cummins’ work including the absence of a timeframe related to ratings of wellbeing, particularly in relation to stressful experiences, the lack of mode of action between specific stressors and their impact on the homeostatic function and that the fact that not all stressors equally impact on wellbeing (see [32,33] where our work addresses some the limitations of Cummins’ model in more detail).

As part of their government reporting requirements the Namoi Catchment Management Authority (CMA) were required to benchmark the wellbeing of their community. The CMA was concerned to benchmark wellbeing amongst specific stakeholders within their communities, and in so doing to consider the potential for individuals to approach or pass through thresholds in wellbeing; and where such risks existed, to use this information to inform their decision as where best they should focus their resources in supporting people to adapt in the face of change. This study was developed to commence this program of inquiry.

## 2. Methods

Three communities were selected for this study, which will be named community A, B and C. Community A is approximately based in the middle of the Murray-Darling Basin where irrigated and dryland cotton are produced. Community A served as the focus of this study with Communities B and C serving as comparative communities; Community B is also a cotton community and is based in the very north of the Basin; and Community C, a citrus and grape growing community, is based in the south of the Basin. To provide a means of continuity in localized data collection, the sampling frame for this study was based upon previous research designs used for Namoi Catchment Management Authority (CMA) projects (see for example [34,35]). These research designs were concerned with the adaptive capacity of key stakeholder groups within the rural communities (specifically farmers, hobby farmers, town residents and change agents), particularly with regards to community involvement in natural resource management. To this end, the sampling for this study was structured to capture the range of views of these stakeholders regarding adaptability and wellbeing in the face of changing climate, water policy and the viability of family farming in agri-dependent communities.

Sample size was determined by the available budget which was sufficient, using computer aided telephone interviewing (CATI), to sample 1,000 people from the Community A district and 1,000 people from the two nominated comparative communities (500 people respectively). The telephone survey was designed to recruit 2,000 people covering the key target audiences on the following basis:

- Primary producers/natural resource managers, n = 400 (Source: a list representing the primary producer and natural resource manager population).
- Community (town dwellers), n = 1,000 (Source: random digit dialling).
- Community (rural dwellers), n = 500 (Source: random digit dialling).
- Change agents, n = 100 (Source: sample from a list).

To participate in the study respondents had to be aged at least 18 years and no older than 75 years of age; no quotas were set by age group. Primary producers were defined as those whose primary occupation and main source of income was some form of primary production (gross agricultural production greater than $5,000 p.a.); sampling quotas were set equally between males and females. Hobby farmers were defined as people living on a small area property outside of town boundaries and whose land holding and scale of enterprise was not large enough to qualify them as a primary producer (gross agricultural production of less than $5,000 p.a.); sampling quotas were set equally between males and females. Town residents were defined as people living in the towns and villages; respondents were representative of the community by gender. Change agents were defined as people engaged in servicing primary producers and land managers through commercial services such as contracting, consulting and sale of goods as well as state, federal and local government employees providing information and advisory services to land managers; no quotas were set for change agents by gender. Primary producers were over-sampled since these people were considered to be the main “client group” for the CMA. The proportion of the sample to be selected from each local government area reflected the demographics of the region and the distribution of primary producers in the local government areas. The survey was fielded in March and April 2012. During this period of time there was a fatal shooting of a local policeman in Community A, while Community B was inundated by flood. All communities had been subject to a severe drought in first decade of this century, particularly during the years 2006–2010.

Table 1 below reports on the actual sample obtained. As is evident from the table, some minor oversampling occurred in Community B (n = 657) and Community C (n = 525). This occurred as a result of the process of random digit dialling which was utilized in this study. Within this design the first four digits of the telephone number are held constant, to capture residents in a given area, while the last four digits are randomly dialled to capture households within the targeted area. As a result of increasing centralization of telephone systems in rural areas, numbers with the same leading four digits may in fact capture residents from both the targeted community and those in neighboring areas. This outcome occurred in this study.

**Table 1:** Sample captured for this survey (%) (n = 2,196).

Area	%
Community A	46
Community B	30
Community C	24
Total	100

**Figure 1:**
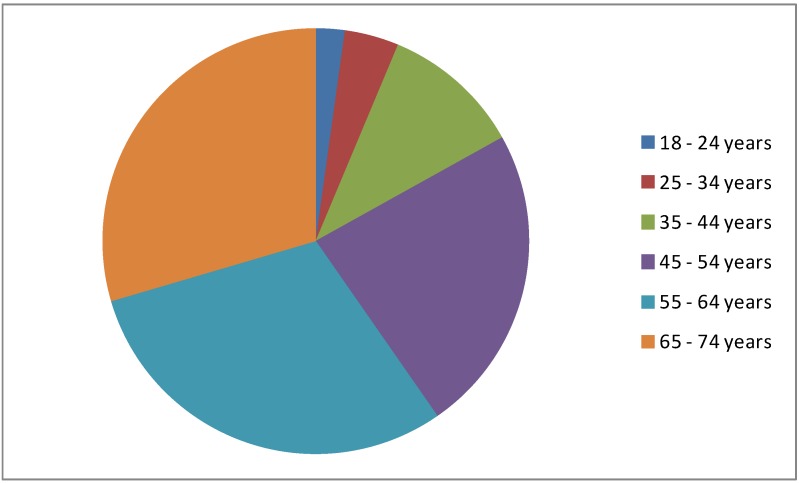
Survey participants by age (n = 2,196).

Quite equal proportions of males and females (49% *versus* 51%) were recruited to participate in the study. Men were slightly over-represented in Community C (Standardized Residual (SR) 22.5) while women were slightly over-represented in Community A (54%) and Community B (58%) (SRs 18.4 and 8.9 respectively) (*Χ*^2^ (2) = 524.1; *p* < 0.001). The age profile of the sample (see Figure 1) was skewed towards the older age group [33]. Participants from Community A were notably older with 63% of participants being aged 55 to 74 years, compared with 52% in Community B and 58% in Community C (*Χ*^2^ (10) = 1,766; *p* < 0.001). The workforce participation rate of these respondents was 59%. Compared to Community B (76%) and Community C (61%), residents in Community A were less likely to be participating in the workforce (54%) (*Χ*^2^ (2) = 978.3; *p* < 0.001).

### Indices and Indicators

This study involved a 20-minute computer-aided telephone interview (CATI) survey of 2,000 respondents from across the three areas of interest. Within this study the notion of adaptive capacity was operationalized as the capacity of individuals and the community to solve the problems they faced, even when under stress. To research these items, the main components of the survey were:

1. Individual adaptive capacity [36].
2. Collective adaptive capacity (ability to work together) including community leadership [37].
3. Social connectedness and social support [38,39].
4. Financial and emotional impacts of significant recent (defined) weather events.
5. Subjective wellbeing index (where scores of less than 6 on a 10-point scale represented a failure in the capacity of the individual to maintain their wellbeing [30,40,41,42,43].
6. Work-life balance [44].
7. Demographic items.
8. Index of cumulative stressful life events occurring in the last 12 months.

Two indices (wellbeing and stress) and five indicators were derived from the survey items. The two indices were:

1. The Subjective Wellbeing Index [40]: for example, ‘I am satisfied with my life as a whole’, scored from 1–10. As needed, this scale may be multiplied by 10 so that it rescales scores to report them between 0 and 100 [40,41].
2. An index of cumulative life stresses made up of items such as ‘Experiencing a natural disaster in the past 12 months, scored from 0–8.

The five indicators were:

1. Individual adaptive capacity: for example, ‘I can solve most problems if I invest the necessary effort’, scored from 0–5 with higher scores indicative of higher perceived levels of individual adaptive capacity.
2. Ability to work together: for example, ‘The people in this community can work together even when it requires more than normal effort’, scored from 0–5 with higher scores indicative of higher perceived levels of collective adaptive capacity.
3. Community connectivity: for example, ‘I always feel that I am an important part of this community’ scored from 0–5 with higher scores indicative of higher perceived levels of community connectivity.
4. Access to social support: for example, ‘I am able to get support from family and friends when needed’, scored from 0–5 with higher scores indicative of higher perceived levels of social support.
5. Efficacy of community leadership: for example, ‘Our Council is not very effective’, scored from 0–5 with higher scores indicative of higher perceived levels of effective local leadership.

The other items in the study were used descriptively. Exploratory factor analysis was used to refine the items to identify the latent contents contained within them (see for example [9]). Details of the psychometric properties of the indices and indicators can be found at [33]. Briefly, factor analysis typically revealed variance explained by the measures in the order of 60–70%; reliability analysis (Cronbach’s alpha) was in the range of 0.6–0.9 for all variables except social capital; mainly due to a lack of items relating to this measure in the study. The descriptive statistics for these measures are reported in Table 2 below.

**Table 2:** Descriptive statistics for the indicators used in this study.

Indicator	Mean	SD	Range	Minimum	Maximum
Deakin wellbeing index	7.8	1.3	9	1	10
Cumulative life stress index	0.76	0.83	7	0	7
Individual adaptive capacity	4.1	0.68	4	1	5
Ability to work together	3.8	0.71	4	1	5
Community connectivity	3.5	0.83	4	1	5
Access to social support	3.8	0.91	4	1	5
Community leadership	3.2	1.04	4	1	5

Two central questions were of interest to this analysis. The first question was concerned with developing an understanding of how useful (from a public health perspective) it was to use a wellbeing approach to understanding how people may be impacted by known, recent stressors, taking into account their capacity to respond in the face of challenges (*i.e.*, their resilience). The second question, following on from the first, was concerned to identify whether specific groups within the community could be identified as lacking resilience, where a lack of resilience was operationalized as lacking both adaptive capacity and wellbeing.

To address the first question a hierarchical linear regression was conducted. The logic of the analysis is theorized from the analysis provided thus far in this paper, while drawing in co-variables known to impact on wellbeing such as gender, age, existing health status and income. In turn we theorized that wellbeing would consequently be influenced by adaptive capacity and social capital. The proposed model (which assumes that wellbeing is positively associated with personal health and social and adaptive capital and negatively associated with shocks and stressors) therefore looks as follows, with subjective wellbeing as the dependent variable:

- Gender and age
- Income
- Financial and emotional impact of major weather events
- Cumulative life stressors
- Self-assessed health
- Individual adaptive capacity
- Collective adaptive capacity
- Social support
- Community connectivity
- Community leadership

Within this paper, only the final table resulting from the hierarchical analysis is presented. Additional tables are available from the authors. The resulting analyses identified that a small group of respondents consistently reported reduced adaptive capacity and wellbeing. Factor-cluster analysis was used to profile members of this group. The analysis was conducted in three steps following the SPSS manual. Hierarchical analysis was used to produce an agglomeration schedule, and from within the graphing of the resulting coefficients four segments were visually evident. The K Means cluster procedure was then run for a four-cluster solution. Within this paper, only the highlights of this analysis are reported, noting that more detailed tables and figures, and further explanation of why specific variables were reported, are available on request, see [33].

**Table 3:** Benchmarking outcomes by communities.

Indicator	Community A	Community B	Community C
Individual adaptive capacity	4.12	4.13	4.07
Ability to work together ^†^	3.74	3.81	3.82
Community connectivity ***	3.38	3.59	3.70
Social support ***	3.85	3.74	3.62
Community leadership	3.17	3.22	3.25
Cumulative stress index ***	0.61	1.14	0.52
Subjective wellbeing scale **	7.74	7.92	7.72

* Statistically significant differences were found between communities; ** *p* < 0.01.; *** *p* < 0.001; ^†^ Approached significance.

## 3. Results

Table 3 compares mean scores of the indicators used in this study by the participating communities. The results show that members of these communities perceive themselves as having high levels of individual and collective adaptive capacity and social capital, reporting approximate mean scores of 4 on a scale of 5. Subjective wellbeing scores (which showed an average of 7.79), were slightly higher than Australia-wide levels of 7.5 [45]. Statistically significant differences were evident between communities with regard to the level of community connectivity, social support, stress and wellbeing.

With the exception of community leadership (25%) and community connectivity (11%), the proportion of respondents who reported scores in the negative range of the indicators (*i.e.*, a score below 3 on a scale of 5, a score below 6 on a scale of 10 on the Subjective Wellbeing Index, and 3 or more recent stressors on the Cumulative Stress Index) was typically less than 10%. Table 7 reports outcomes on the indicators used in this study by key stakeholder groupings. Slightly higher proportions of community members reported lower levels of community connectivity (10.7%) or lacking confidence in their community leadership (25.1%), while 8.4% reported lower levels of wellbeing. Statistically significant differences were observed between groups with regard to community connectivity, social support, community leadership and cumulative stressors (see Table 4).

**Table 4:** Benchmarking outcomes by stakeholder groups.

	Primary producer	Town resident	Hobby farmer	Change agent
Individual adaptive capacity	4.08	4.12	4.12	4.15
Ability to work together	3.78	3.77	3.78	3.81
Community connectivity ***	3.63	3.46	3.57	3.58
Social support ***	3.52	3.90	3.64	3.82
Community leadership ***	3.04	3.29	3.12	3.10
Cumulative stress index ***	0.89	0.66	0.79	0.79
Deakin wellbeing scale	7.86	7.78	7.79	7.79

*** Statistically significant differences were found between communities *p* < 0.001.

Examining the data for differences which may be of practical significance, change agents reported higher levels of recent cumulative life stressors than did town residents. Some notable differences in the indicators were observed between members of the respective stakeholder groupings. Notably, primary producers (SR 3.0) were more likely to report greater connectivity than other stakeholders while town residents were more likely to report lesser connectivity (SR 3.0) (*X*^2^ (12) = 26.2; *p* < 0.01). Town residents were more likely (SR 4.8) to report the highest perceptions of social support while primary producers reported the least (SR −4.3) (*X*^2^ (12) = 113.1; *p* < 0.001). Town residents (SR 2.0) were more likely to rate community leadership highly while primary producers were less likely to highly rate community leadership (−2.5) (*X*^2^ (12) = 34.6; *p* < 0.001). Almost half the stakeholders (43.5%) reported no significant stressors in the past twelve months. Primary producers (SR 2.6) were more likely to report having experienced three or more significant stressors in the past twelve months, while town residents were less likely to report such stressors (SR −2.5) (*X*^2^ (9) = 37.8; *p* < 0.001).

### 3.1. Examining the Relationship between Adaptive Capacity and Subjective Wellbeing

Table 5 examines correlations between the respective variables concerned with adaptive capacity, social capital and subjective wellbeing. Notable correlations include subjective wellbeing with individual adaptive capacity (0.331), collective adaptive capacity (ability to work together) (0.318) and community connectivity (0.335). The variables of individual adaptive capacity and collective adaptive capacity were correlated (0.193), and collective adaptive capacity and social support were correlated (0.208). Collective adaptive capacity and community leadership demonstrated the highest correlation in this study (0.368). Stressors showed the least degree of correlation with the other variables.

**Table 5:** Correlations between items measuring adaptive capacity and wellbeing.

	Individual adaptive capacity	Ability to work	Community connectivity	Social support	Community leadership	Cumulative stressors	Deakin wellbeing scale
Individual adaptive capacity	1	0.193	0.190	0.069	0.098	0.003 ^†^	0.331
Ability to work	0.193	1	0.334	0.208	0.368	−0.031 ^†^	0.318
Community connectivity	0.190	0.334	1	0.132	0.156	−0.032 ^†^	0.335
Social support	0.069	0.208	0.132	1	0.223	−0.062	0.180
Community leadership	0.098	0.368	0.156	0.223	1	−0.063	0.213
Cumulative stressors	0.003 ^†^	−0.31 ^†^	−0.032 ^†^	−0.062	0.063	1	−0.055
Deakin wellbeing scale	0.331	0.318	0.335	0.180	0.213	−0.055	1

N = 2,196; all correlations statistically significant at 0.05 (2 tailed) except for correlations for some cumulative stressors as denoted ^†^.

Hierarchical linear regression was conducted to examine the extent to which the various indicators of adaptive capacity, taking into account stressors, were associated with subjective wellbeing. The statistical results of this analysis can be seen in Table 6 below.

**Table 6:** Associations between adaptive capacity and human wellbeing (including self-reported health).

Model	*B*	Standard error	Standardized beta coefficients	*t*	Sig.
Constant	1.162	0.224		5.189	0.000
Age group	0.131	0.018	0.131	7.327	0.000
Gender	0.130	0.046	0.050	2.844	0.004
Household income	0.054	0.010	0.101	5.656	0.000
Self-reported health	0.432	0.023	0.347	19.107	0.000
Individual adaptive capacity	0.378	0.035	0.198	10.93	0.000
Community connectivity	0.261	0.029	0.167	8.871	0.000
Social support	0.127	0.026	0.089	4.977	0.000
Ability to work together	0.225	0.036	0.123	6.184	0.000
Community leadership	0.097	0.024	0.077	4.093	0.000

The model, which was statistically significant at each step in the analysis, accounted for 36% (F (9, 2099) = 139.3; *p* < 0.001) of the variance. Statistics concerning multi-collinearity were well within acceptable limits (e.g., variance inflation factor < 5). Considering the variables of interest, self-assessed health was most highly associated with subjective wellbeing (accounting for 27% of variance) followed by individual adaptive capacity (15%), community connectivity (13%), collective adaptive capacity (the ability to work together) (10%) and age (10%). Financial stressors were associated with wellbeing, but were superseded in the model first by the experience of cumulative recent stressors, and in turn health.

Table 7 below presents the Anova table for the K means cluster analysis. This table shows that subjective wellbeing, the ability to work together and community leadership are the most influential variables in the cluster solution.

**Table 7:** Anova table for the cluster analysis.

	Cluster		Error		F	Sig.
	Mean Square	df	Mean Square	df		
Individual adaptive capacity	193.990	3	0.736	2,192	263.619	0.000
Ability to work together	293.673	3	0.599	2,192	489.909	0.000
Community connectivity	194.571	3	0.735	2,192	264.695	0.000
Social support	219.411	3	0.701	2,192	312.961	0.000
Community leadership	265.525	3	0.638	2,192	416.204	0.000
Deakin Wellbeing index	294.910	3	0.598	2,192	493.365	0.000

The F tests should be used only for descriptive purposes because the clusters have been chosen to maximize the differences among cases in different clusters. The observed significance levels are not corrected for this and thus cannot be interpreted as tests of the hypothesis that the cluster means are equal.

### 3.2. People Lacking Resilience in the Face of Vulnerabilities

**Figure 2:**
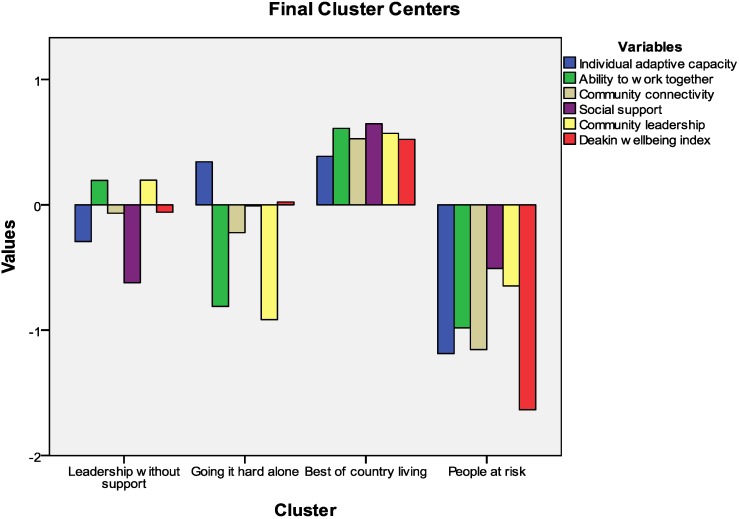
Visual presentation of the 4-cluster solution.

Figure 2 provides a visual presentation of the 4-cluster solution using standardized scoring. Within this figure, a score of zero represents an average score. Scores greater than zero represent a result which is above average, and scores less than zero represent a result which is below average.

Based on the attributes of each segment, the cluster groupings were labeled:

1. *Leadership without support* (30%).
2. *People going it hard alone* (22%).
3. *The best of country living* (37%).
4. *People at risk* (11%).

Data describing the key descriptors of the cluster group memberships can be found in Table 8. A description of the key attributes of the respective segments follows.

*Leaders without support* (30%) reported above-average perceptions of the efficacy of community leadership and the capacity to work together, but below-average perceptions of individual adaptive capacity, connectivity, collective adaptive capacity and wellbeing. Primary producers were well represented in this segment, as were women and people living in Community C. Approximately two-thirds of this segment was in some form of full time paid employment. They enjoyed good job security and felt they had employment options should they lose their current job. 

**Table 8:** Key descriptive statistics describing segment attributes.

	Leadership without support (%)	Going it hard alone (%)	Best of country living (%)	People at risk (%)
Female *	56	45	52	50
Income over $120,000 *	7	13	10	4
Aged > 55 years *	59	56	57	49
In paid employment/business *	62	68	60	52
Work in agriculture *	46	40	36	34
Job security (strongly agree) *	31	40	50	14
Able to find another job locally	26	32	35	23
Involved in church groups *	28	20	28	19
Involved in sports clubs *	32	36	37	24
Agree climate change is affecting community *	40	36	41	34
3 or more stressors in last year	2.6	3.7	2.4	5.3
Subjective wellbeing less than threshold *	2.9	3.8	1.3	55.7

* *p* < 0.05 or greater

Notably more than one-third would retire if they lost their current job. Climate events, particularly droughts, had financially as well as emotionally impacted on this segment. Nonetheless they were slightly over-represented among respondents who agreed that climate change was affecting their community. They reported being in good but not excellent health. They were highly involved in church and sporting groups.

*People going it hard alone* (22%) reported above-average levels of individual adaptive capacity and slightly above-average wellbeing, while reporting below-average scores on the other indicators. Respondents from Community C were under-represented in this segment while respondents from Community A were slightly over-represented. Members of this segment were more commonly men, and aged over 55 years. Agriculture was the main employer. Members of this segment were also more likely to be living in shared accommodation and a disproportionate number reported earning incomes in highest income bracket (<$120,000 per annum). If they lost their jobs one-third would retire, but a large proportion (12%) considered that they would need to leave the area to find further employment. Members of this segment were less likely to be involved in church activities. Taken together these indicators paint a picture of capable individuals who lack community connectivity and support and who are vulnerable to change, particularly in agriculture.

People enjoying the *best of country living* (37%) reported above-average adaptive capacity, connectivity, social support, ability to work together, community leadership and wellbeing. They were more likely to be town residents. While no single community stood out, Community B trended towards being predominant in this segment. There were no significant differences in this segment by gender. However, this segment did enjoy an over-representation of younger people. Two-thirds of this segment is in some form of full time paid employment; they reported the highest levels of job security, and were commonly working in agriculture, health services or education. Unlike other segments, these respondents were very positive about their capacity to find another job in the area, if they needed to. The segment had an under-representation of tradespeople. Members of this segment reported being less financially or emotionally impacted by drought and flood events. They were over-represented among respondents who agreed that climate change was affecting their community. They reported the lowest mean level of cumulative stressors (0.7). They reported the best health of all the segments and they also reported a higher proportion of people in the highest income level (although this difference was not statistically significant). They were more likely to live in households with no children and they reported above-average levels of involvement in churches and sports clubs.

*People at risk* (11%) constituted the most at-risk segment and reported experiencing below-average perceptions of their adaptive capacity, the ability to work together, community connectivity and leadership, social support and subjective wellbeing. Members of this segment were equally represented across respondent types as well as the participating communities. Members of this segment were the most vulnerable in the workforce. They reported the lowest rate of full-time employment and were notably over-represented amongst the casual workforce. They were also over-represented amongst those with TAFE and equivalent qualifications. As well, they were more likely to be physically unable to work, or to be unemployed. Those in work reported the lowest levels of job security and were more likely to report that work and family life interfered with each other and that they were dissatisfied with the amount of leisure time they had. If they lost their job they expected that it would be more difficult than others to find another job locally. They reported the highest cumulative life stressors and the worst health. They were more likely to be in households of single people aged over 30 years and were over-represented within the two lowest income groupings. They were less socially connected, being more likely to not be involved in either church or sporting groups.

## 4. Discussion and Conclusions

The findings in this study are circumscribed by the fact that it was based on a cross-sectional research design. Additional modeling (e.g., structural equation modeling) and longitudinal studies are required to verify the insights developed from this initial project. Longitudinal studies are important because a cross-sectional analysis does not enable one to know which sequence of events actually led to, or was protective of wellbeing. Similarly, we do not know what factors led to placing some people in the at risk group. Poorer health outcomes were part of their profile, but we cannot know from this study design whether these conditions were endogenous in nature, or indeed if health outcomes were brought on by the stressors to which they have been subjected. It is feasible that some of the people in the at risk group are vulnerable as a result of factors which have nothing to do with their environmental settings. Moreover, it may be that a sequence of events is of concern (e.g., social stressors arising from environmental stressors, which in turn produce health effects that diminish wellbeing). While the feasibility of such a sequence can be modeled using structural equation modeling, testing of a causal sequence requires longitudinal analysis. The study was also limited by the fact that quota-based sampling were utilized and as such the sample is not representative of the populations as a whole; notably, young people 18–25 years were under-represented in the study while farmers were over-represented.

Members of rural communities have often felt that the social aspects of managing change are important, but have had difficulties operationalizing such concepts into applied research [10]. This study makes headway in addressing this gap in the literature. The study found that a majority of the members of the study communities enjoy a high level of wellbeing and adaptive capacity. The study found that indicators of individual (0.331) and collective (0.318) adaptive capacity were correlated with subjective wellbeing, while cumulative stressors correlated poorly. Subjective wellbeing was associated with adaptive capacity, taking into account gender, age income, a variety of stressors, existing self-assessed health and social capital. The segmentation analysis made it possible to observe a group of at risk individuals whose wellbeing scores were both well below the average and well below the threshold level proposed by Cummins. The profile provided for this group also made it evident just how vulnerable members of this group were. The results of this analysis also provided support for Cork *et al.* [29] contention that adaptive capacity and wellbeing are as much distributed within communities as they are between communities. This result is especially important because present approaches to adaptation policy tend to focus on place, rather than seeking to identify those sub-groups and individuals within a community who are most likely to be vulnerable to change. A place-based focus can assume that everyone within a given area may be equally affected by external events and in so doing does not provide for differences in pre-existing physical, personal or economic resources. As Hogan *et al.* [46] demonstrated, farmers working within similar geographically defined spaces may in fact have access to quite differing qualities of farm resource to work with (rain, for example, may fall literally on one side of a hill and not the other).

Several preliminary insights are evident from this initial study. First, focusing on the capacity of individuals to work with others and to adapt to change, serve as important strategies in maintaining wellbeing in communities under stress. Second, given the sequence in which the stress-related variables were eliminated from the regression model, it is feasible that while stressors do impact on wellbeing, their effects are manifested in health outcomes and adaptive capacity (albeit the results of the latter analysis, being so similar, are not reported here). Assuming that this insight is further validated, it lends support to recent insights in the literature which propose that a key strategy for building adaptive capacity is enabling people to come to appreciate that they have in fact been affected by stressors of change processes [47,48]. Third, wellbeing may serve as a useful and parsimonious proxy measure for resilience and adaptive capacity. These insights are supported by the findings of the segmentation analysis. Given the association between adaptive capacity and wellbeing, and the differences which exist between sub-groups within the study communities on these indicators, the results of this study promote the usefulness of identifying the nature of vulnerability within a community as much as across communities. Given the presence of such differences, strengthening strategies need to be tailored to the needs of specific sub-sectors; a one-size fits all approach would not be appropriate.
